# Robust passive and active efflux of cellular cholesterol to a designer functional mimic of high density lipoprotein

**DOI:** 10.1194/jlr.M054635

**Published:** 2015-05

**Authors:** Andrea J. Luthi, Nicholas N. Lyssenko, Duyen Quach, Kaylin M. McMahon, John S. Millar, Kasey C. Vickers, Daniel J. Rader, Michael C. Phillips, Chad A. Mirkin, C. Shad Thaxton

**Affiliations:** * Department of Chemistry Northwestern University, Evanston, IL 60208; ††† International Institute for Nanotechnology, Northwestern University, Evanston, IL 60208; † Lipid Research Group, Division of Gastroenterology, Hepatology, and Nutrition, Children’s Hospital of Philadelphia Perelman School of Medicine at the University of Pennsylvania, Philadelphia, PA 19104; § Division of Translational Medicine and Human Genetics, Department of Medicine, Perelman School of Medicine at the University of Pennsylvania, Philadelphia, PA 19104; ** Robert H. Lurie Comprehensive Cancer Center, Northwestern University, Chicago, IL 60611; †† Walter S. and Lucienne Driskill Graduate Training Program in Life Sciences, Northwestern University, Chicago, IL 60611; §§ Department of Urology, Feinberg School of Medicine, Northwestern University, Chicago, IL 60611; §§§ Simpson Querrey Institute for BioNanotechnology and Medicine, Northwestern University, Chicago, IL 60611; *** Department of Medicine, Vanderbilt University School of Medicine, Nashville, TN 37232

**Keywords:** ATP-binding cassette transporter A1, atherosclerosis, macrophages, cholesterol efflux, ATP-binding cassette transporter G1, scavenger receptor class B type I

## Abstract

The ability of HDL to support macrophage cholesterol efflux is an integral part of its atheroprotective action. Augmenting this ability, especially when HDL cholesterol efflux capacity from macrophages is poor, represents a promising therapeutic strategy. One approach to enhancing macrophage cholesterol efflux is infusing blood with HDL mimics. Previously, we reported the synthesis of a functional mimic of HDL (fmHDL) that consists of a gold nanoparticle template, a phospholipid bilayer, and apo A-I. In this work, we characterize the ability of fmHDL to support the well-established pathways of cellular cholesterol efflux from model cell lines and primary macrophages. fmHDL received cell cholesterol by unmediated (aqueous) and ABCG1- and scavenger receptor class B type I (SR-BI)-mediated diffusion. Furthermore, the fmHDL holoparticle accepted cholesterol and phospholipid by the ABCA1 pathway. These results demonstrate that fmHDL supports all the cholesterol efflux pathways available to native HDL and thus, represents a promising infusible therapeutic for enhancing macrophage cholesterol efflux. fmHDL accepts cholesterol from cells by all known pathways of cholesterol efflux: unmediated, ABCG1- and SR-BI-mediated diffusion, and through ABCA1.

HDL performs a critical role in the pathogenesis of coronary artery disease (CAD) and acute coronary syndrome (1). The inverse association between HDL cholesterol (HDL-C) mass and CAD risk prompted the active investigation of HDL metabolism in the context of CAD (2). However, recent human genetic studies and clinical trials suggest that HDL-C mass and CAD are not causatively linked (3, 4). Epidemiological observations instead point to macrophage HDL-C flux as a causative atheroprotective factor (5). Macrophage HDL-C flux is the net transport of cholesterol from macrophages to the liver by HDL (5, 6). Unlike HDL-C mass, macrophage HDL-C flux is a dynamic parameter not readily amenable to direct quantification in human subjects (5). Nevertheless, HDL-C efflux capacity (HDL-CEC) from macrophages, i.e., the amount of macrophage cholesterol that HDL is capable of accepting, can be assessed ex vivo using immortalized or primary macrophage cells (7, 8). HDL-CEC varies from individual to individual and is inversely correlated with carotid intima-media thickness, angiographic CAD, and acute coronary syndrome independent of HDL-C mass (9–12). These findings suggest that high HDL-CEC and, by extension, HDL-C flux are essential for cardiovascular health.

Given its involvement in CAD pathogenesis, HDL-CEC is an attractive target for an antiatherogenic therapeutic intervention with the goal of increasing the transport of cholesterol from macrophages to the liver (13–15). Cholesterol acceptance by HDL is interwoven with HDL particle formation and maturation (6, 16). In the formation step, cellular lipids, including cholesterol and lipid-poor apo A-I, assemble into discoidal HDL particles in a process mediated by ABCA1. In the maturation step, discoidal HDL binds LCAT, which esterifies free cholesterol associated with the lipoprotein. Formed cholesteryl ester enters the HDL particle core and brings about a shape change to a sphere of increasing size (5). Esterification by LCAT creates a concentration gradient that promotes unmediated and ABCG1- and scavenger receptor class B type I (SR-BI)-facilitated diffusion of cholesterol from cells to discoidal and spherical HDL (5, 17). In clinical trials, infusion of natural or synthetic precursors of mature HDL, such as apo A-I, apo A-I analogs, or reconstituted discoidal HDL (rdHDL) into the human body has increased HDL-CEC and reduced plaque size and atheroma volume, but has also induced unfavorable changes in the blood lipid profile (13–15, 18, 19). In particular, administration of rdHDL has raised the levels of VLDL cholesterol (VLDL-C) and LDL cholesterol (LDL-C), both potent atherogenic factors that may offset any positive effect that accrues from elevated HDL-CEC (19–21). Thus, further investigation into alternative forms of synthetic HDL is warranted.

Previously, we reported the synthesis of a nanostructure designed to mimic the ability of HDL to accept cellular cholesterol (22, 23). This functional mimic of HDL (fmHDL) contains a gold nanoparticle (Au NP) core, which serves as a template for the adsorption of apo A-I and assembly of mixed phospholipid layers. In shape and size, fmHDL resembles mature spherical HDL. A survey of different sizes and surface chemistries found that fmHDL particles with a 6 nm diameter Au NP core, one to three molecules of apo A-I modified to contain sulfhydryl groups (apo A-I-SH), and a phospholipid bilayer [fmHDL (apo A-I-SH)] exhibit high binding affinity for a cholesterol analog and support cellular cholesterol efflux (23). Here, we present a detailed characterization of cholesterol efflux from immortalized cells and primary macrophages to fmHDL (apo A-I-SH) and an fmHDL formulation that contains unmodified apo A-I [fmHDL (apo A-I)]. The results show that fmHDL (apo A-I) accepts cellular cholesterol not only by unmediated and ABCG1- and SR-BI-facilitated diffusion, but also by the critically important antiatherogenic ABCA1 pathway and thus, may provide a means to increase HDL-CEC and alleviate CAD.

## MATERIALS AND METHODS

### Preparation of HDL, apo A-I, [^3^H]apo A-I, and apo A-I-SH

HDL (1.065 g/ml < d < 1.21 g/ml) and HDL_3_ (1.125 g/ml < d < 1.21 g/ml) were isolated by sequential ultracentrifugation from a pool of normolipidemic frozen human plasma as described by Havel, Eder, and Bragdon (24), stored at 4°C in KBr, and used within 4 weeks of preparation. Apo A-I was either purchased (Meridian Life Science) or isolated from HDL (25). Apo A-I-SH was prepared from apo A-I by converting primary amines to sulfhydryls with Traut’s reagent (2-iminothiolane; Thermo Fisher Scientific) (26). Traut’s reagent was dissolved in water at 2 mg/ml (14 mM) immediately before use. Apo A-I (1 ml) at 1 mg/ml was diluted with 500 μl of a buffer containing PBS (Life Technologies) and 4 mM EDTA (Ambion) at pH 8.0. Apo A-I has 22 primary amines (27), and thus, a 20-fold molar excess of Traut’s reagent (50.4 μl at 14 mM) was added to the apo A-I solution. The protein was incubated at room temperature for 1 h with gentle shaking. Five milliliter Zeba spin desalting columns (Thermo Fisher Scientific) were used to purify apo A-I-SH. [^3^H]apo A-I (∼1 μCi/mg) was prepared by reductive methylation of apo A-I with [^3^H]formaldehyde (American Radiolabeled Chemicals) in the presence of sodium cyanoborohydride followed by dialysis in 0.1 M NaCl (28). Protein content was quantified by either the Lowry method or absorbance at 280 nm using extinction coefficients 0.86 ml/mg·cm for HDL_3_ (29) and 1.13 ml/mg·cm or 32,430 M^−1^cm^−1^ for apo A-I.

### Synthesis of fmHDL

fmHDL was synthesized using citrate-stabilized colloidal Au NPs (Ted Pella). Transmission electron microscopy (TEM) analysis of the Au NPs sold as “5 nm in diameter” revealed that the diameter of these nanoparticles was actually 6 ± 1 nm (23). A 5-fold molar excess of apo A-I or apo A-I-SH (see above) was combined with the Au NPs, and the apolipoprotein/Au NP mixture was incubated on a shaker at 130 rpm and room temperature for 1 h. Then, the mixture was diluted with ethanol 0.2-fold. Ethanol (Sigma Aldrich) was used to solubilize the phospholipids and limit formation of phospholipid vesicles and micelles (30, 31). 1,2-Dipalmitoyl-*sn*-glycero-3-phosphoethanolamine-N-[3-(2-pyridyldithio)propionate] (sodium salt) (PDP PE) and 1,2-dipalmitoyl-*sn*-glycero-3-phosphocholine (DPPC) (both from Avanti Polar Lipids) were dissolved in ethanol before each particle preparation. PDP PE and DPPC were sequentially added to the mixture, both in 250-fold molar excess relative to the starting amount of Au NPs. The mixture was returned to the shaker and incubated overnight (∼12 h). For fmHDL without apo A-I [fmHDL (no apo A-I)], the synthesis was performed by the same procedure except the addition of apo A-I was omitted.

A KrosFlo Research II*i* tangential flow filtration system (Spectrum Laboratories) was employed to purify fmHDL by diafiltration, a combination of filtration and dialysis in which impurities are removed as the sample passes over a semipermeable membrane (32). A MidiKros module (Spectrum Laboratories) with a 50 kDa modified polyether sulfone membrane was used for diafiltration of fmHDL (apo A-I) and fmHDL (apo A-I-SH). fmHDL (no apo A-I) was purified with a 30 kDa modified polyether sulfone membrane MidiKros module. The general diafiltration parameters were a pump speed of 30 ml/min, shear of 5,000–7,000 s^−1^, and pressure of 8–12 PSI. fmHDL samples were concentrated, washed with ∼40 ml of water, and collected. New modules were used for each sample preparation. After each sample purification, the tangential flow filtration system was rinsed with water until the eluant remained clear and colorless, and then sequentially washed with 1 M NaOH twice, water three times, 30% isopropanol twice, water three times, and stored in 0.1 M NaOH. The sample volumes were further reduced under a stream of N_2_ to reach Au NP concentrations of ∼1–5 μM. The Au NP concentration of fmHDL was determined by measuring A_524_ (extinction coefficient 9.696 × 10^6^ M^−1^cm^−1^; Ted Pella) on a Cary 5000 UV-Vis-NIR spectrophotometer (Agilent Technologies), as Au colloid absorbs light strongly at ∼520 nm due to the surface plasmon resonance (33). Based on TEM and dynamic light scattering (DLS) data (see below), each fmHDL particle contains one Au NP. Thus, fmHDL concentrations are the same as the Au NP concentrations. fmHDL was stored at 4°C and used within 3–7 days of synthesis.

### Fluorescence-based measurement of apolipoprotein concentration in fmHDL

The strong absorbance of UV and visible light by Au NPs and of UV light by the PDP PE 2-pyridyl group rendered the common absorbance-based protein quantification methods unreliable and precluded direct determination of apo A-I or apo A-I-SH concentration in each fmHDL sample. A fluorescence-based method was used to quantitate apolipoprotein content in representative fmHDL (apo A-I) and fmHDL (apo A-I-SH) samples, as previously described (23). Apo A-I was labeled with AlexaFluor® 488 (Life Technologies) in accordance with the dye manufacturer’s instructions with one modification. During column chromatography to purify dye-labeled apo A-I, the column pieces were glued together, and a stream of N_2_ was passed through the column to propel the sample along. Dye-labeled apo A-I was further modified with Traut’s reagent (see above) to obtain apo A-I-SH. The molar concentrations of apo A-I-Alexa 488 and apo A-I-SH-Alexa 488 were determined by absorbance as recommended by the dye manufacturer. Standard curves of apolipoprotein concentration versus Alexa 488 fluorescence were constructed separately for apo A-I-Alexa 488 and apo A-I-SH-Alexa 488 by serial dilution of the dye-labeled apolipoproteins with PBS/80 mM KCN (Sigma-Aldrich)/0.01% Tween 20 (Sigma-Aldrich) in a 96-well plate. Fluorescence was measured at 520 nm (490 nm excitation) using a BioTek Synergy H4 multi-mode microplate reader. fmHDL particles were synthesized with the same apo A-I-Alexa 488 (two different preparations) or apo A-I-SH-Alexa 488 (three different preparations) and purified by diafiltration (see above). fmHDL concentrations were determined based on Au NP absorbance at 524 nm. Aliquots of the fmHDL samples were diluted with water and PBS/80 mM KCN/0.01% Tween 20 (∼60 mM KCN/0.008% Tween 20 final concentrations) to 10 nM fmHDL in a 96-well plate and read with the plate reader. KCN and Tween 20 were added to dissolve the Au NPs that would otherwise quench fluorescence and to solubilize the proteins and phospholipids, respectively. Fluorescence measurements were converted to molar concentrations of apo A-I-Alexa 488 and apo A-I-SH-Alexa 488 using the standard curves.

### Gas chromatography-mass spectrometry

The palmitate content of fmHDL preparations was measured at the Metabolic Tracer Resource (Penn Institute of Diabetes, Obesity, and Metabolism). Fifty microliter subsamples of 4.71 μM fmHDL preparations were combined with heptadecanoic acid (>99%, Sigma-Aldrich) as an internal standard (1.5 μM final concentration) and 200 μl of 0.3 N KOH in ethanol and incubated at 70°C for 30 min to saponify phospholipid. Palmitic acid standards ranging from 0 to 200 μM were made using a gravimetrically prepared stock solution of palmitic acid (>99%, Sigma-Aldrich). Fifty microliters of each standard were combined with heptadecanoic acid internal standard (1.5 μM final concentration). Following saponification of fmHDL samples, fatty acids in samples and standards were esterified with 14% boron trifluoride in methanol and analyzed on an Agilent 7890A/5975C series GC/MSD (Agilent Technologies) using electron ionization. Palmitate peak abundance was expressed relative to the internal standard and quantified using a standard curve (micromoles of palmitate versus the ratio of the peak abundances of *m/z* 270:284).

### fmHDL particle stoichiometry

The average number of apo A-I or apo A-I-SH molecules per fmHDL particle was determined using fmHDL preparations made with apo A-I-Alexa 488 or apo A-I-SH-Alexa 488 (see above). The molar concentration of Au NPs in the preparations was calculated by the formula: A_524_ × dilution factor/9.696 × 10^6^ M^−1^. The molar concentrations of apo A-I-Alexa 488 and apo A-I-SH-Alexa 488 were derived from standard curves of apolipoprotein concentration (calculated by the formula: [A_280_ − (A_494_ × 0.11)] × dilution factor/32,430 M^−1^, as recommended by the dye manufacturer) versus Alexa 488 fluorescence. The moles of apolipoprotein were divided by the moles of Au NPs to calculate the average number of apo A-I and apo A-I-SH molecules per Au NP and thus, per fmHDL. In a different approach, fmHDL (apo A-I) was synthesized with [^3^H]apo A-I. The molar concentration of Au NP was calculated as above, whereas the molar content of apo A-I was derived from a standard curve of apo A-I concentration (determined as A_280_ × dilution factor/32,430 M^−1^) versus [^3^H]apo A-I counts per minute values. To determine the average number of phospholipid molecules per fmHDL, fmHDL was prepared normally, the Au NP concentration was measured as described above, and the molar concentration of palmitate in fmHDL was determined using gas chromatography-mass spectrometry (see above). Because both PDP PE and DPPC contain two palmitoyl acyl chains, the palmitate molar concentration was divided by two to calculate the molar concentration of phospholipid. The molar concentration of phospholipid was divided by the molar concentration of Au NP to determine the average number of phospholipid molecules per fmHDL. In a radiolabel-based approach, fmHDL (apo A-I) was made with L-α-dipalmitoyl,[choline-methyl-^3^H]phosphatidylcholine (PerkinElmer), and the molar concentration of DPPC in the fmHDL samples was calculated from a standard curve of [^3^H]DPPC concentration versus [^3^H]DPPC counts per minute. All stoichiometric data are provided in **Table 1**.

**TABLE 1.** Hydrodynamic diameter of fmHDL and its precursors during synthesis and fmHDL particle stoichiometry and zeta potential

Nanostructure	Hydrodynamic Diameter (nm)	Zeta Potential (mV)	Apo A-I Molecules per Particle	Phospholipid Molecules per Particle	DPPC Molecules per Particle
Au NPs	7 ± 1	—	—	—	—
Au NP + apo A-I	10 ± 1	—	—	—	—
Au NP + apo A-I + phospholipid	23 ± 1	—	—	—	—
fmHDL (apo A-I) purified	13 ± 1	−58 ± 9	3.1–3.4*^a,b^*	350 ± 10	N/D
			3.3 ± 0.2*^c^*		
fmHDL (apo A-I-SH) purified	13 ± 1	−50 ± 10	4.0 ± 0.4*^b^*	300 ± 100	N/D
fmHDL (no apo A-I) purified	15 ± 1	−50 ± 10	—	360 ± 20	190 ± 20

All numbers are rounded to two significant figures and are the mean of three independent preparations ± SD; N/D, not determined.

a These values are based on two independent preparations.

b These values were derived using fluorescently labeled apo A-I and apo A-I-SH (see Materials and Methods).

c These values were obtained using [^3^H]apo A-I (see Materials and Methods).

### Apolipoprotein cross-linking on fmHDL

fmHDL samples were diluted to 50 μg/ml apolipoprotein with water/10× sodium phosphate buffer (10 mM sodium phosphate final, pH 7.4). Bis[sulfosuccinimidyl] suberate (BS^3^) (Thermo Scientific) was added to the final concentration of 2.5 mM, and cross-linking reactions proceeded for 30 min at room temperature. To generate an apo A-I oligomer ladder, lipid-free apo A-I was dialyzed in 10 mM sodium phosphate buffer (pH 7.4), diluted with the same buffer to 0.5 mg/ml, and cross-linked with 0.25 mM BS^3^ at room temperature for 30 min. The 0.5 M Tris base (45 mM final) was added to stop the reactions. Cross-linked products were resolved on 4–20% Tris-glycine SDS-PAGE gels and visualized via immunoblotting with a goat polyclonal anti-apo A-I antibody (NB400-147; Novus Biological).

### Transmission electron microscopy

A pair of tweezers was used to hold a 200 mesh copper grid covered with a carbon film (Electron Microscopy Sciences), while a 5 μl drop of 500 nM fmHDL was pipetted onto the grid’s carbon side. The drop was allowed to adsorb to the grid for 15 min. Then the excess solution was wicked away with filter paper, and the grid was left to dry for five more minutes. A 40 μl drop of 2% (w/w) uranyl acetate (UA) in water was pipetted onto a small piece of parafilm. The grid was floated on the UA drop with the sample side down for 7 min and then placed on a piece of filter paper with the sample side up to dry. The UA solution was shielded from light to minimize stain degradation. TEM images were taken with a FEI Tecnai Spirit G2 transmission electron microscope operating at 120 kV. Proteins and phospholipids have low electron density and require staining for TEM imaging. In UA-stained samples, apolipoproteins and phospholipids are visible as white rings around the electron dense Au NP.

### Dynamic light scattering and circular dichroism

Size and zeta potential were determined by DLS with a Zetasizer Nano ZS (Malvern Instruments). Measurements were taken with the following parameters: 50–100 nM of fmHDL in water, material refractive index of 0.30, and absorbance of 0.234. At least five measurements were averaged for each sample, and at least three different fmHDL preparations were measured. The number function was used to determine the hydrodynamic diameter.

For circular dichroism (CD), 1.5 μM of apo A-I or 500 nM of fmHDL (three different preparations) were used. Background was subtracted for each measurement. The background for apo A-I was water (three samples) or 1× PBS with 4 mM EDTA at pH 8.0 (one sample). The solvents did not affect the apo A-I spectra. fmHDL (no apo A-I) was used as the background for apo A-I-containing fmHDL to subtract the CD signal from the Au NPs and phospholipids. Measurements were done with a Jasco J-815 CD spectrophotometer. Three different databases (SELCON3, CDSSTR, and CONTIN) were used to analyze the CD spectra, and the average of the results from the three databases is reported as the percentage of residues having a particular secondary structure.

### Cell culture

Baby hamster kidney (BHK)-ABCA1 and BHK-ABCG1 cells were kindly provided by the late Dr. Jack Oram (34). BHK-SR-BI cells are described by Vickers et al. (35). Fu5AH cells were a kind gift of Dr. George Rothblat (36). Cells were routinely passaged at 1/10 dilution twice a week and grown in DMEM supplemented with 10% FBS and 50 μg/ml gentamicin at 37°C in 5% CO_2_. Primary resident peritoneal mouse macrophages were obtained from 2- to 4-month-old all male or all female C57BL/6 mice (37). Peritoneal exudate cells in PBS were centrifuged at 400 *g* for 10 min at 4°C. Supernatant was discarded, and the cell pellet was resuspended in cold DMEM supplemented with 10% FBS and 50 μg/ml gentamicin. Cells were counted with a hemocytometer, plated at an approximate density of 4 × 10^5^ cells/well in 24-well plates, and allowed to recover overnight before further experimentation.

### fmHDL and HDL_3_ concentrations used in cholesterol efflux assays

As mentioned above, protein measurement methods based on absorbance were not suitable for apo A-I quantification in fmHDL preparations. The apolipoprotein content of fmHDL was calculated by the equations: apo A-I micrograms per milliliter = [fmHDL] × 3.3 × 28,080/10^6^ and apo A-I-SH micrograms per milliliter = [fmHDL] × 4 × 28,080/10^6^, where [fmHDL] is the concentration of fmHDL calculated from A_524_ (see above), 3.3 and 4 are the number of apo A-I and apo A-I-SH, respectively, molecules per fmHDL (see above and Table 1), 28,080 is apo A-I mass in Daltons and 10^6^ is a unit adjustment parameter. The fmHDL concentration for the BHK-ABCA1 and BHK-ABCG1 cholesterol efflux assays was 0.118 μM for all three types of fmHDL, or 0.118 × 3.3 × 28,080/10^6^ ≈ 11.0 μg/ml in terms of apo A-I in fmHDL (apo A-I) and 0.118 × 4 × 28,080/10^6^ = 13.3 μg/ml in terms of apo A-I-SH in fmHDL (apo A-I-SH). Four-fold higher amounts (0.472 μM) were used in the SR-BI-mediated cholesterol efflux assays in BHK-SR-BI and Fu5AH cells, and 0.236 μM (2×) amounts were used for cholesterol efflux assays in mouse macrophages. All protein in fmHDL was apolipoprotein. The concentration of HDL_3_ in terms of the total protein content was matched to the apo A-I concentration of fmHDL (apo A-I).

### Cell cholesterol efflux assays

BHK-ABCA1, BHK-ABCG1, and BHK-SR-BI cells were seeded at 1/10 dilution from confluent flasks into 24-well plates in DMEM supplemented with 10% FBS and 50 μg/ml gentamicin (500 μl of medium/well) and allowed to attach overnight. On day 2, the medium was changed to DMEM supplemented with 2.5% FBS, 50 μg/ml gentamicin, and 0.5–1.0 μCi/ml [1,2-^3^H(N)]cholesterol (PerkinElmer), and the cells were incubated for 24 h to label the intracellular pools of cholesterol. On day 3, the medium was changed to DMEM supplemented with 0.2% BSA (fraction V, fatty acid free; EMD Millipore), 50 μg/ml gentamicin, and either 10 nM mifepristone (Sigma-Aldrich) or vehicle, and the cells were incubated overnight for 16–20 h. On day 4, the medium was changed to DMEM supplemented with 50 μg/ml gentamicin and either 10 nM mifepristone or vehicle, with or without a cholesterol acceptor (fmHDL, HDL_3_, or apo A-I), and the cells were incubated for 4 h (2 h in the case of BHK-SR-BI). fmHDL suspended in water comprised 10% of the cholesterol efflux medium. To account for any effects of medium dilution, water was added to 10% to acceptor-free and HDL_3_- or apo A-I-containing media. The latter two cholesterol acceptors were suspended/dissolved in TBS. To account for any effects of TBS on cholesterol efflux, an equivalent amount of TBS was added to the fmHDL and acceptor-free media. At the end of the incubation period, cell media were collected and filtered through 96-well filter plates (EMD Millipore). A 100 μl aliquot of each sample was read in a scintillation counter. Cell lipids were extracted with hexane-isopropanol (3:2, v/v), the solvent was evaporated, and the lipids were read in a scintillation counter. [^3^H] counts in the medium were multiplied by five, divided by the sum of [^3^H] counts in the medium and in the cells, and multiplied by 100 to calculate the percentage of total cellular cholesterol released to the cholesterol acceptors.

Fu5AH cells were plated and labeled as detailed above. On day 3, the cells were incubated in DMEM supplemented with 0.2% BSA and 50 μg/ml gentamicin for 3–4 h, treated with 1 M block lipid transport-1 (BLT-1) (Sigma-Aldrich) or DMSO for 1–1.5 h in DMEM supplemented with 50 μg/ml gentamicin, and then exposed to fmHDL, HDL_3_, or acceptor-free medium for 2 h in DMEM supplemented with 50 μg/ml gentamicin in the presence of the inhibitor or vehicle. The cell media and cells were analyzed as described above.

Primary macrophages, seeded as outlined above, were labeled with 0.5 μCi/ml [1,2-^3^H(N)]cholesterol in DMEM supplemented with 2.5% FBS and 50 μg/ml gentamicin overnight. On day 3, the cells were exposed to 200 μg/ml LDL in DMEM supplemented with 0.2% BSA and 50 μg/ml gentamicin overnight. On day 4, the cells were treated with probucol (Sigma-Aldrich), BLT-1, or vehicles for 1–1.5 h and then exposed to fmHDL in the presence of the same inhibitors for 4 h, in both cases in DMEM supplemented with 50 μg/ml gentamicin. Cell media and cells were analyzed as described above. To introduce probucol into the medium, DMEM supplemented with 50 μg/ml gentamicin in a 15 ml conical tube was heated to 37°C in a water bath. Probucol (20 mM) in ethanol was pipetted into the preheated medium while it was vigorously vortexed.

### Large-scale ABCA1-mediated lipid efflux and gel filtration analysis of fmHDL

BHK-ABCA1 cells (three T75 flasks per cholesterol acceptor) were plated at 1/10 dilution from a confluent culture and allowed to attach overnight in DMEM supplemented with 10% FBS and 50 μg/ml gentamicin. On day 2, the cells were labeled with 0.06 μCi/ml [4-^14^C]cholesterol (PerkinElmer) alone or 0.06 μCi/ml [4-^14^C]cholesterol and 1.3 μCi/ml [methyl-^3^H]choline chloride (PerkinElmer) in DMEM supplemented with 2.5% FBS and 50 μg/ml gentamicin overnight. On day 3, the cells were treated with 10 nM mifepristone or vehicle for 16–20 h in DMEM supplemented with 0.2% BSA (fraction V) and 50 μg/ml gentamicin. Then the cells were exposed to fmHDL (apo A-I), fmHDL ([^3^H]apo A-I), apo A-I, or acceptor-free medium in DMEM supplemented with 50 μg/ml gentamicin for 8 h. fmHDL ([^3^H]apo A-I) was also added to DMEM supplemented with 50 μg/ml gentamicin in T75 flasks that did not contain cells (no cells control). Cell media were collected, filtered through 0.45 μm polyvinylidene difluoride (PVDF) membrane filter units (EMD Millipore), reduced in volume 20-fold from 30 to 1.5 ml using Amicon Ultracel-10K centrifugal filters (EMD Millipore), and stored at 4°C for further analysis. A 1 ml aliquot of the 20-fold concentrated cell medium was resolved into 1 ml fractions on a calibrated HiLoad 16/60 Superdex 200 gel filtration column (GE Healthcare). TBS (pH 7.4) was the mobile phase. Five hundred microliters of each fraction were transferred to a cuvette to measure A_523_. The remainder was combined with 5 ml of ScintiVerse BD cocktail (Fisher Scientific) and read in a scintillation counter. [^3^H] and [^14^C] counts were adjusted for the energy emission spectra overlap. The gel filtration column was washed between runs with 30% isopropanol and 1 M NaOH, as recommended by the column manufacturer. The following standards (Sigma-Aldrich) were used to calibrate the column: cytidine, Vt; thyroglobulin, 17.0 nm; apoferritin, 12.2 nm; lactic dehydrogenase, 8.16 nm; BSA, 7.1 nm; carbonic anhydrase, 4.4 nm; blue dextran, Vo.

## RESULTS

### fmHDL synthesis

**Fig. 1.**
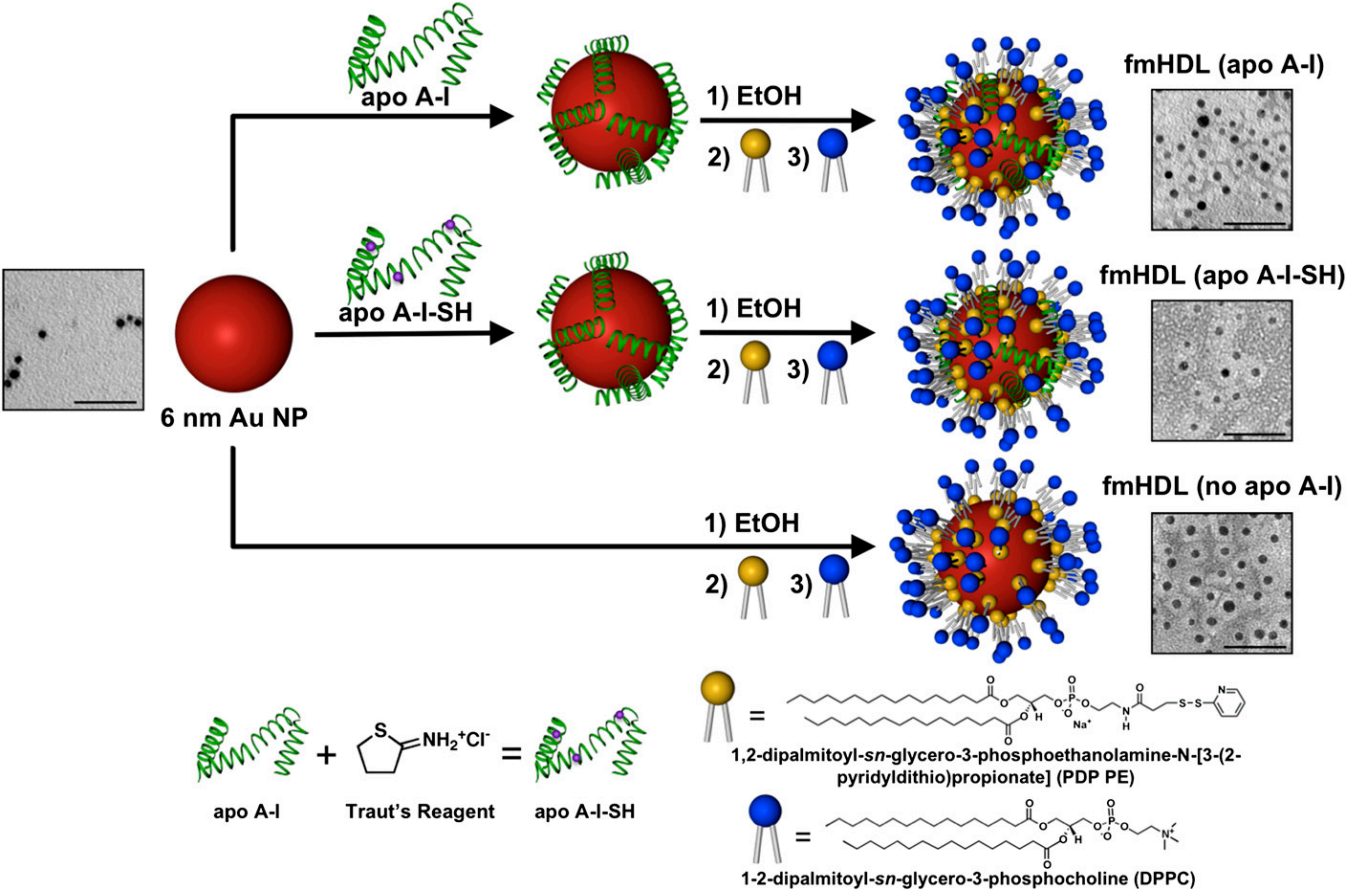
Schematic of fmHDL synthesis and TEM images of unmodified Au NPs and fmHDL particles. Au NPs were first combined with a 5-fold molar excess of apo A-I or apo A-I-SH in an aqueous buffer and incubated for 1 h. Apo A-I-SH was synthesized by modifying apo A-I with Traut’s reagent. The Au NP/apolipoprotein mixture was diluted with ethanol (EtOH) to a final ethanol content of approximately 20%. PDP PE and then DPPC (both in 250-fold molar excess over Au NPs) were added, and the mixture was incubated overnight. This procedure generated fmHDL (apo A-I) and fmHDL (apo A-I-SH). fmHDL (no apo A-I) was synthesized by the same method except apolipoprotein was not added. In the TEM images, the protein-lipid or lipid-alone bilayer is visible as a white ring around the electron dense Au NP. Unmodified Au NPs lack such rings. Scale bars are 50 nm.

Self-assembly of organosulfur compounds such as thiols (RSH) and disulfides (RSSR) into monolayers on the surface of Au NPs offers the opportunity to synthesize structures with a wide range of properties and potential medical applications (33). We have used PDP PE, a phospholipid that carries a disulfide group, to synthesize Au NP-based structures, termed fmHDLs, which are designed to exhibit the key antiatherogenic property of native HDL, i.e., its ability to accept macrophage cholesterol (1, 15). The overall fmHDL synthesis is outlined in **Fig. 1** and detailed in the Materials and Methods. The synthesis procedure yielded three types of particle: fmHDL (apo A-I), fmHDL (apo A-I-SH), and fmHDL (no apo A-I). In our working model of the fmHDL nanostructure, an Au NP core is enveloped in a bilayer of either phospholipid and apolipoprotein, as in fmHDL (apo A-I) and fmHDL (apo A-I-SH), or phospholipid alone, as in fmHDL (no apo A-I) (Fig. 1). The organosulfur molecule PDP PE, which self-assembles on the Au NP surface, preferentially forms the inner monolayer of the lipid-protein bilayer. The acyl chains of PDP PE extend away from the Au NP and hydrophobically interact with the acyl chains of DPPC, which preferentially makes up the outer monolayer of the bilayer. Although apo A-I-SH may be tethered to the gold surface by the sulfhydryl modification, unmodified apo A-I is likely held on the fmHDL particle by the same electrostatic and hydrophobic interactions with the lipid bilayer that act between apo A-I and lipids in native HDL.

The association of apolipoproteins and phospholipid with Au NPs was monitored by measuring hydrodynamic diameter of the intermediate nanostructures with DLS (Table 1). The nanostructures that assembled in the Au NP and apo A-I mixture were larger than the original Au NPs (7 ± 1 nm vs. 10 ± 1 nm; mean ± SD). Addition of PDP PE and DPPC led to a further increase in size to 23 ± 1 nm. Purification removed loosely associated components and reduced the nanostructure size. The diameters of fully-prepared fmHDL (apo A-I), fmHDL (apo A-I-SH), and fmHDL (no apo A-I) particles were 13 ± 2, 13 ± 1, and 15 ± 1 nm, respectively. Given that the thickness of a lipid bilayer, including the inner and outer monolayers and outer hydration layer, is approximately 4.3 nm (38), our working model predicts that the hydrodynamic diameter of fmHDL should be 14.6 nm (6 nm gold core + 8.6 nm encasing lipid bilayer), which is very close to the experimentally determined value.

### fmHDL stability, stoichiometry, and structural properties

In negatively-stained TEM images of fmHDL, the apolipoproteins and phospholipids were visible as white rings around the electron dense Au NP; in unmodified Au NPs, these rings were absent (Fig. 1). Regardless of the presence or absence of apolipoprotein, fmHDL had a negative zeta potential in water with values ranging from −50 ± 14 mV to −58 ± 9 mV (Table 1). Zeta potential is the electrokinetic potential at the outer layer of solvent ions that are tightly associated with the nanoparticle surface (39). The highly negative zeta potential values indicate that fmHDL is stable in water, as zeta potentials greater than 30 mV or less than −30 mV signify colloidal stability (39). However, fmHDL (no apo A-I) exhibited a tendency to aggregate in DMEM. To detect DMEM-induced aggregation, fmHDL particles were suspended in DMEM/50 μg/ml gentamicin, filtered through 0.45 μm PVDF filter units, measured at A_523_, and incubated for 4 h at 37°C. Then, the particles were filtered again through 0.45 μm PVDF filters and measured at A_523_. The fmHDL (no apo A-I) A_523_ values after the incubation were 30–40% (n = 2) lower than before. The reduction in absorption was due to retention of the aggregates on the filter membrane. The before and after A_523_ measurements for fmHDL (apo A-I) and fmHDL (apo A-I-SH) were identical. This difference in particle aggregation with and without apolipoprotein suggests that its presence promotes fmHDL stability.

**Fig. 2.**
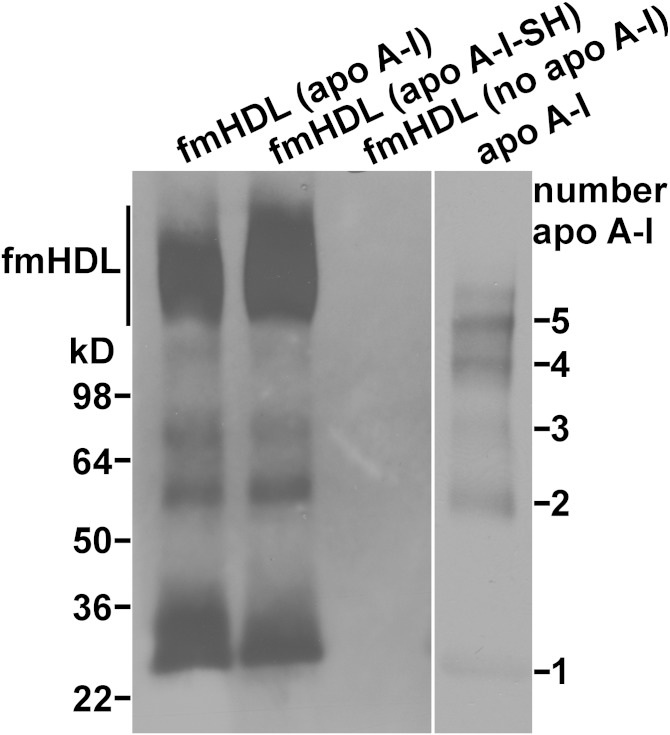
Cross-linking analysis of fmHDL apolipoprotein. fmHDL (apo A-I), fmHDL (apo A-I-SH), and fmHDL (no apo A-I) were cross-linked with BS^3^. The apolipoprotein cross-link products were resolved on a 4–20% SDS PAGE gel and visualized by apo A-I immunoblotting. An oligomer ladder was prepared with lipid-free apo A-I (apo A-I lane) for cross-link product identification. fmHDL migrated on the PAGE gel as a clearly visible band and were detectible by apo A-I immunoblotting (marked as fmHDL on the left).

Dye-labeled apo A-I and apo A-I-SH were used to determine the average number of apolipoprotein molecules per fmHDL particle (see Materials and Methods and Table 1). Based on this method, fmHDL (apo A-I) and fmHDL (apo A-I-SH) contained 3.1–3.4 and 4.0 ± 0.4, respectively, molecules of apolipoprotein per particle. In a different approach, fmHDL (apo A-I) was prepared with [^3^H]apo A-I, and the average number of apo A-I molecules per particle was determined using the [^3^H]apo A-I specific activity. This method showed that fmHDL (apo A-I) contained 3.3 ± 0.2 molecules of apo A-I per particle. Apolipoproteins on fmHDL were also cross-linked with BS^3^, an amine-to-amine protein cross-linker, and then resolved on an SDS PAGE gel and visualized by an anti-apo A-I antibody (40). While the products corresponding to two and three cross-linked apo A-I or apo A-I-SH molecules were distinctly visible, the product corresponding to four cross-linked apolipoprotein molecules was at the limit of detection indicating that four was the highest number of apolipoprotein molecules per fmHDL particle (**Fig. 2**). Together, these data suggest that fmHDL (apo A-I) and fmHDL (apo A-I-SH) contained two to four molecules of apolipoprotein per particle.

As both PDP PE and DPPC carry palmitoyl acyl chains, gas chromatography-mass spectrometry was used to determine the amount of palmitate on fmHDL and to calculate the number of phospholipid molecules per fmHDL particle (Table 1). fmHDL (apo A-I), fmHDL (apo A-I-SH), and fmHDL (no apo A-I) contained 350 ± 14, 300 ± 98, and 360 ± 15 phospholipid molecules per fmHDL, respectively. To determine the amount of DPPC per fmHDL, fmHDL (no apo A-I) was prepared with DPPC spiked with [^3^H]DPPC, and the number of DPPC molecules per fmHDL was calculated using a [^3^H]DPPC standard curve. fmHDL (no apo A-I) contained 190 ± 20 molecules of DPPC per fmHDL. The larger number of DPPC than PDP PE molecules per particle, 190 versus 170 (360 minus 190), is in line with our working model of fmHDL, which suggests that DPPC preferentially forms the more voluminous outer monolayer, while PDP PE preferentially comprises the less voluminous inner monolayer (Fig. 1).

Sulfhydryl addition may constrain the structural conformation of apo A-I and account for any differences in functionality between fmHDL (apo A-I) and fmHDL (apo A-I-SH). CD was used to characterize the secondary structure of apolipoprotein in lipid-free and fmHDL-associated forms (**Table 2**). A significantly higher percentage of lipid-free apo A-I-SH assumed an α-helical conformation compared with unmodified lipid-free apo A-I. Nonetheless, apo A-I-SH was indistinguishable from apo A-I in its ability to sustain cellular cholesterol efflux from BHK-ABCA1 cells via ABCA1 (data not shown). These observations suggest that incorporation of sulfhydryl groups onto apo A-I impacts its lipid-free conformation but not the ability to promote efflux. Apo A-I and apo A-I-SH on fmHDL were both about 85% α-helical. This percentage of α-helicity is similar to what is typically observed for apo A-I associated with larger-sized reconstituted spherical HDL (25).

**TABLE 2.** Structural properties of apo A-I and apo A-I-SH in lipid-free and fmHDL-associated forms

	α-Helix (%)	β-Sheet (%)	Turns (%)	Unordered (%)
Lipid-free apo A-I	27 ± 2	19 ± 3	22 ± 4	30 ± 3
Lipid-free apo A-I-SH	40 ± 2	15 ± 2	17 ± 1	28 ± 2
fmHDL (apo A-I)	85 ± 5	2 ± 1	6 ± 6	11 ± 5
fmHDL (apo A-I-SH)	86 ± 5	2 ± 1	6 ± 6	10 ± 7

All numbers are rounded to two significant figures. Values are the mean of three independent preparations ± SD.

### fmHDL accepts cholesterol by unmediated and ABCG1-facilitated diffusion

Mature spherical HDL acquires macrophage cholesterol by unmediated and ABCG1- and SR-BI-facilitated diffusion; it can also release lipid-poor apo A-I, which accepts cholesterol via ABCA1 (6, 16, 17). To study these pathways individually, we have taken advantage of BHK cells engineered to express one of the three cell cholesterol release proteins under the control of a mifepristone-inducible promoter. BHK-ABCG1 cells inducibly express human ABCG1 (34). Cholesterol efflux from these cells in the absence of mifepristone represents simple diffusion alone, whereas it is a sum of both simple and ABCG1-facilitated diffusion in the presence of mifepristone (16).

BHK-ABCG1 cells treated with mifepristone or vehicle were incubated with one of the three types of fmHDL or with HDL_3_. fmHDL samples were added to efflux media to obtain the final particle concentration of 0.118 μM. Because of the difference in the number of apo A-I molecules per particle (Table 1), the final protein concentration was 11.0 μg/ml and 13.3 μg/ml for fmHDL (apo A-I) and fmHDL (apo A-I-SH), respectively (Materials and Methods). Selection of an rdHDL preparation as a positive control would be arbitrary, because rdHDL formulations differ from one another and from native discoidal HDL in the lipid composition and functionality (19–21, 25, 34, 40). Native discoidal HDL cosegregates with the HDL_3_ fraction of spherical HDL, but cannot be further purified in preparative amounts (41). Thus, HDL_3_ was selected as a positive control because it is diverse, readily available, and similar to the apolipoprotein-containing fmHDL in terms of the number of apo A-I molecules per particle, i.e., 3–5 in HDL_3_ (27) versus 3.1–4.4 in fmHDL (Table 1). HDL_3_ was added to the same total protein concentration, 11.0 μg/ml, as fmHDL (apo A-I).

**Fig. 3.**
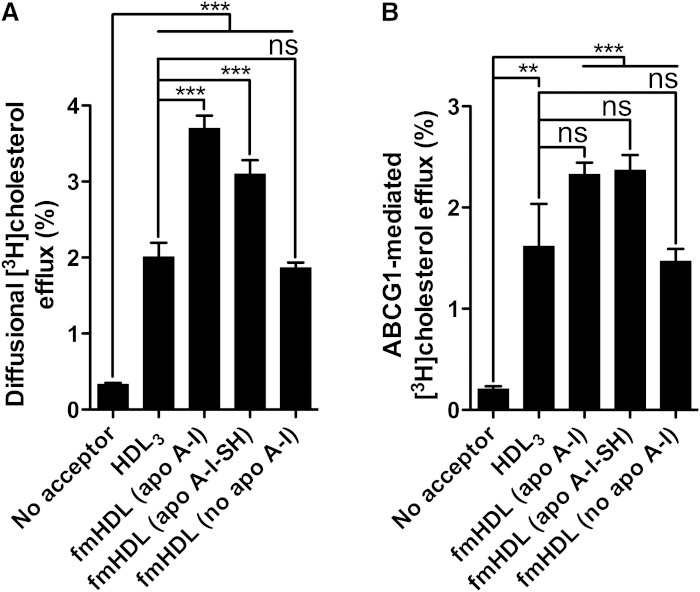
Cellular cholesterol efflux by simple and ABCG1-facilitated diffusion to fmHDL and HDL_3_. A: Cholesterol efflux from BHK-ABCG1 cells by simple diffusion (i.e., in the absence of mifepristone treatment to induce ABCG1 expression). B: To calculate ABCG1-facilitated cholesterol efflux, the average percent of unmediated efflux to each acceptor in (A) was subtracted from the corresponding individual cholesterol efflux values in cells expressing ABCG1 (i.e., treated with mifepristone). Values are the mean ± SEM (n = 9). ***P < 0.001; **P < 0.01; ns, not significant.

All of the acceptors acquired cholesterol by unmediated diffusion (**Fig. 3A**). fmHDL (apo A-I) and fmHDL (apo A-I-SH) obtained significantly more cholesterol by this pathway than fmHDL (no apo A-I) or HDL_3_. The percent efflux values were 3.4 ± 0.5, 2.8 ± 0.5, 1.5 ± 0.2, and 1.7 ± 0.6 for fmHDL (apo A-I), fmHDL (apo A-I-SH), fmHDL (no apo A-I), and HDL_3_, respectively (mean adjusted for efflux to medium without a cholesterol acceptor ± SD). All of the cholesterol acceptors also acquired cholesterol by ABCG1-facilitated diffusion (Fig. 3B). fmHDL (apo A-I) and fmHDL (apo A-I-SH) tended to acquire more cholesterol through ABCG1 (2.1 ± 0.3% and 2.2 ± 0.5%, respectively) compared with fmHDL (no apo A-I) and HDL_3_ (1.3 ± 0.4% and 1.4 ± 1.3%, respectively), but the differences were not statistically significant. Thus, relative to HDL_3_, all fmHDL particles ensure robust cellular cholesterol efflux by unmediated and ABCG1-facilitated diffusion.

### fmHDL accepts cholesterol by the SR-BI-mediated pathway

**Fig. 4.**
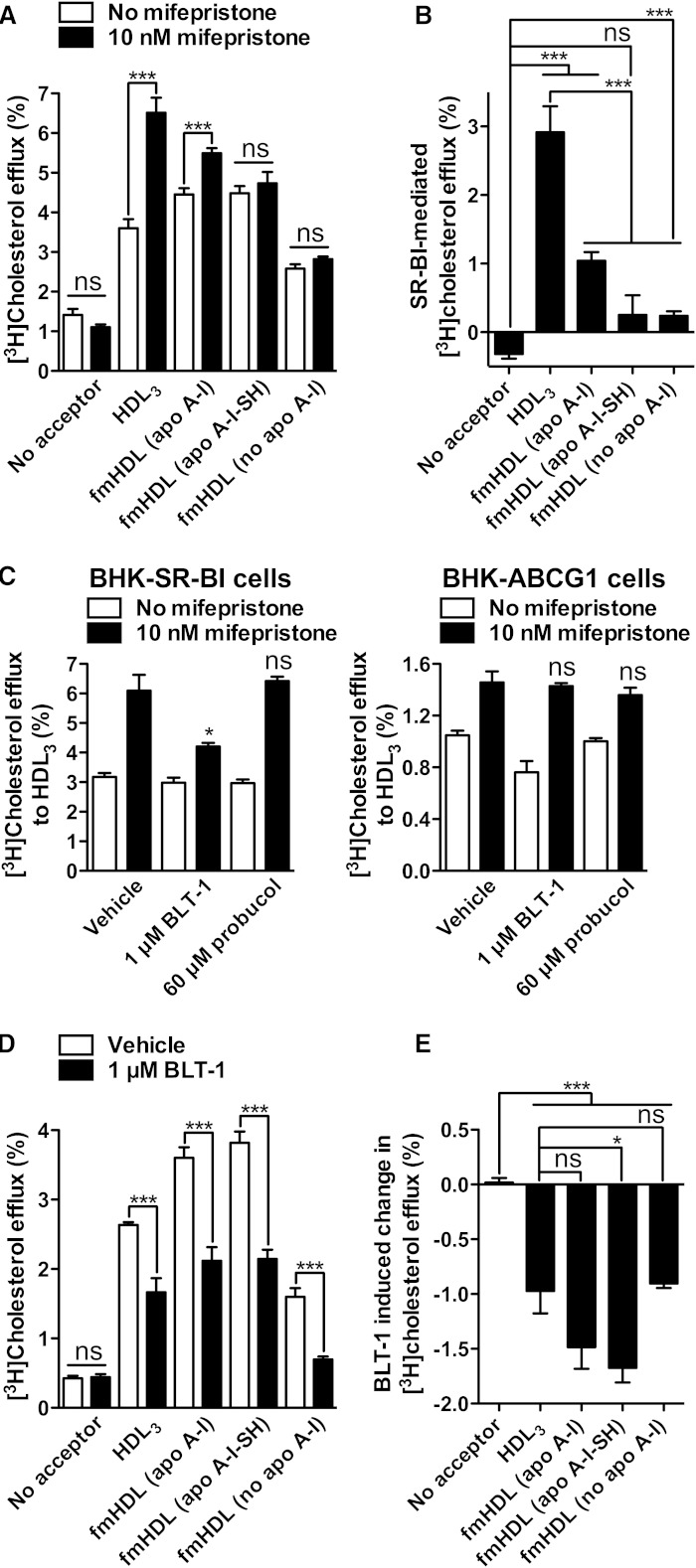
Cholesterol efflux through the SR-BI pathway. A: Cholesterol efflux from BHK-SR-BI cells to the cholesterol acceptors of interest when SR-BI is not expressed (no mifepristone) or when SR-BI is expressed (10 nM mifepristone). B: SR-BI-specific cholesterol efflux was obtained by subtracting the average percent of unmediated (i.e., in the absence of mifepristone) efflux from individual cholesterol efflux values in cells expressing SR-BI. C: Specificity of BLT-1 and probucol inhibitory activity. BLT-1 inhibited SR-BI-mediated efflux in BHK-SR-BI cells, but did not inhibit the ABCG1 pathway in BHK-ABCG1 cells. Probucol affected neither the SR-BI- nor the ABCG1-mediated pathways in BHK-SR-BI and BHK-ABCG1 cells, respectively. D: Cholesterol efflux from Fu5AH cells with and without 1 μM BLT-1. The same amounts of the acceptors were added as in (A). E: SR-BI-specific cholesterol efflux from Fu5AH cells was obtained by subtracting the average percent of cholesterol efflux in the presence of BLT-1 from the corresponding individual cholesterol efflux values in vehicle-treated cells. Greater inhibition of cholesterol efflux with BLT-1 indicates a more robustly functioning SR-BI pathway. Values are mean ± SEM; n = 9 for BHK-SR-BI cells; n = 6 for Fu5AH cells. ***P < 0.001; *P < 0.1; ns, not significant.

SR-BI-specific cholesterol efflux was studied using two different cell lines. BHK-SR-BI cells express human SR-BI under the control of a mifepristone-inducible promoter, whereas rat hepatoma Fu5AH cells express endogenous SR-BI constitutively (35, 36). Low passage number BHK-SR-BI cells treated with vehicle or mifepristone were incubated with one of the three types of fmHDL or with HDL_3_ at concentrations four times higher than the amounts used to support ABCG1-facilitated efflux. In comparison with the ABCG1 and ABCA1 routes, the SR-BI pathway requires higher levels of the acceptor for efficient cellular cholesterol release (36). The lipoprotein concentrations selected for SR-BI efflux (0.472 μM fmHDL and 44 μg/ml HDL_3_) reflected a balance between the need for higher acceptor levels and the technical ability to synthesize fmHDL at high concentrations. All of the acceptors robustly supported unmediated cholesterol diffusion from BHK-SR-BI cells, as was observed for BHK-ABCG1 cells (Fig. 3A versus no mifepristone values in **Fig. 4A**). Expression of SR-BI due to the addition of mifepristone resulted in a significant increase in cholesterol efflux to HDL_3_ (Fig. 4B). SR-BI-mediated efflux to fmHDL (apo A-I) was much lower in magnitude though still significant, whereas SR-BI expression only trivially elevated cholesterol release to fmHDL (apo A-I-SH) and fmHDL (no apo A-I).

Cholesterol efflux values from vehicle-treated Fu5AH cells encompass cholesterol released by unmediated diffusion, the SR-BI pathway, and potentially other pathways, whereas the loss of cholesterol efflux due to treatment with BLT-1 is attributed to suppression of solely SR-BI-mediated efflux (36, 42). BLT-1 is a specific inhibitor of SR-BI. It inhibited SR-BI-mediated cholesterol efflux in BHK-SR-BI cells, but did not significantly affect the ABCG1 pathway in BHK-ABCG1 cells (Fig. 4C). BLT-1 is not an inhibitor of the ABCA1 pathway either (42). Fu5AH cells were exposed to the same concentrations of fmHDL or HDL_3_ as in the case of BHK-SR-BI cells. Treatment of Fu5AH cells with BLT-1 resulted in a significant decrease in efflux to all of the cholesterol acceptors (Fig. 4D). The decreases were very similar, ranging from 0.9 ± 0.1% and 1.0 ± 0.5% for fmHDL (no apo A-I) and HDL_3_, respectively, to 1.5 ± 0.5% and 1.7 ± 0.3% for fmHDL (apo A-I) and fmHDL (apo A-I-SH), respectively (Fig. 4E). Together the studies in BHK-SR-BI and Fu5AH cells suggest that the ability of fmHDL to support SR-BI-mediated cholesterol efflux is cell type-specific.

### Apolipoprotein-containing fmHDL supports active cholesterol efflux by the ABCA1-mediated pathway

**Fig. 5.**
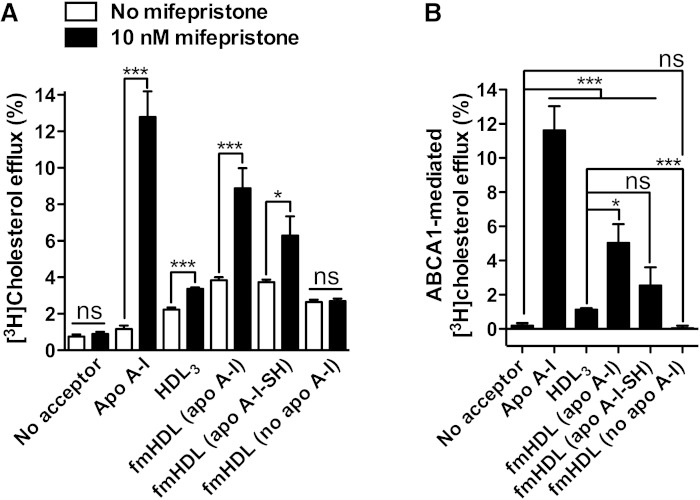
Active cholesterol efflux by the ABCA1 pathway. A: Cholesterol efflux from BHK-ABCA1 cells to the cholesterol acceptors of interest when ABCA1 is not expressed (no mifepristone) or is expressed (10 nM mifepristone). B: ABCA1-specific efflux was calculated by subtracting the average no-mifepristone efflux from the corresponding individual efflux values for cells treated with mifepristone. Values are mean ± SEM; n = 12 for fmHDL and apo A-I; n = 6 for HDL_3_. ***P < 0.001; *P < 0.1; ns, not significant.

BHK-ABCA1 cells treated with or without mifepristone were incubated with one of the three types of fmHDL, HDL_3_, or apo A-I. fmHDL and HDL_3_ were added in the same amounts as in the case of efflux from BHK-ABCG1 cells. Apo A-I was added at 10 μg/ml. Apo A-I did not obtain cholesterol by unmediated diffusion, whereas the other acceptors supported this pathway to an extent similar to that observed with BHK-ABCG1 and BHK-SR-BI cells (Fig. 3A and no mifepristone values in Fig. 4A and **Fig. 5A**). Expression of ABCA1 resulted in 11.6 ± 4.7% increase in cholesterol efflux to apo A-I (Fig. 5B). There were also significant increases in efflux to fmHDL (apo A-I), fmHDL (apo A-I-SH), and HDL_3_: 5.0 ± 3.8%, 2.5 ± 3.7%, and 1.1 ± 0.2%, respectively. No increase in efflux to fmHDL (no apo A-I) was observed, suggesting that apo A-I is essential for the ABCA1 pathway to function, as is expected (16). These results indicate that apo A-I-containing fmHDL can support active ABCA1-mediated cholesterol release.

### fmHDL (apo A-I) releases lipid-poor apo A-I minimally but supports ABCA1-mediated phospholipid efflux

**Fig. 6.**
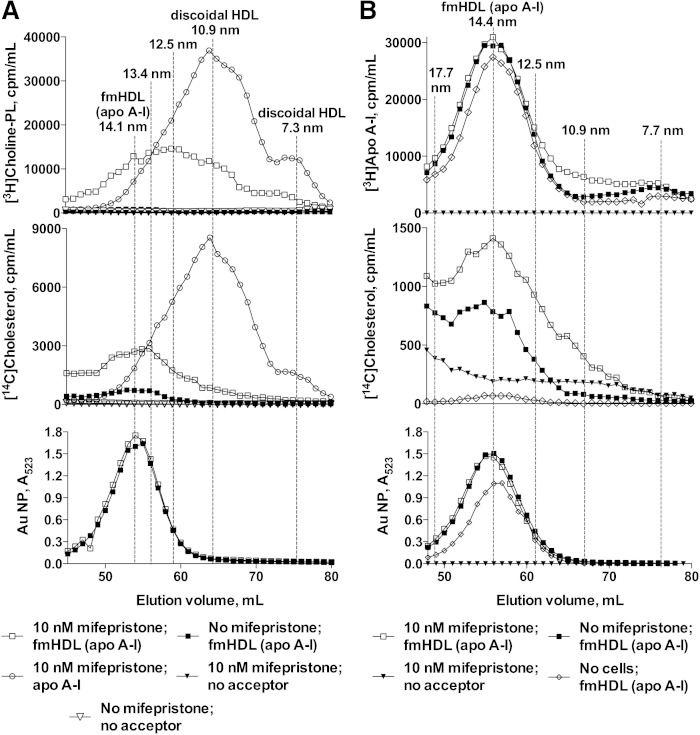
Gel filtration chromatography of fmHDL (apo A-I) exposed to BHK-ABCA1 cells with and without ABCA1 expression. A: BHK-ABCA1 cells labeled with [^14^C]cholesterol and [^3^H]choline-phospholipid were treated with vehicle or mifepristone and incubated with fmHDL (apo A-I) or lipid-free apo A-I. Cell media were concentrated and analyzed on a gel filtration column. fmHDL (apo A-I) particles were detected by measuring A_523_. B: BHK-ABCA1 cells were labeled with [^14^C]cholesterol, treated with vehicle or mifepristone, and exposed to fmHDL prepared with [^3^H]apo A-I. A no-cells control was generated by incubating fmHDL ([^3^H]apo A-I) in cell medium in tissue culture flasks without cells. The gel filtration columns used in A and B differed in the void volume. Particle sizes are approximate.

ABCA1-mediated cholesterol efflux to fmHDL was further investigated by gel filtration in conjunction with cell lipid radiolabeling (40). BHK-ABCA1 cells were incubated with radiolabeled cholesterol and choline, treated with vehicle or mifepristone, and exposed to fmHDL (apo A-I) (0.118 μM), lipid-free apo A-I (10 μg/ml), or medium without a cholesterol acceptor. The efflux media were concentrated and resolved on a gel filtration column. [^14^H]cholesterol and [^3^H]choline-phospholipid in discoidal HDL particles that formed from lipid-free apo A-I and cell lipids eluted in broad peaks centered at the 10.9 and 7.3 nm size fractions (**Fig. 6A**). fmHDL (apo A-I) (indentified by A_523_ from the Au NP) eluted as a broad peak centered at the 14.1 nm mark, a size value similar to the one obtained by DLS (Fig. 6A and Table 1). For fmHDL exposed to vehicle-treated cells, the A_523_ peak completely overlapped with the [^14^H]cholesterol peak, while a corresponding [^3^H]choline-phospholipid peak was absent. This indicates that only simple diffusion of cholesterol from cells to fmHDL (apo A-I) took place in the absence of ABCA1. For fmHDL exposed to ABCA1-expressing cells, the A_523_ peak overlapped with an enlarged [^14^H]cholesterol peak and a prominent [^3^H]choline-phospholipid peak, indicating that ABCA1 supported cholesterol and phospholipid efflux to fmHDL (apo A-I). However, the center of the fmHDL phospholipid peak (12.5 nm fraction) eluted after the center of the fmHDL A_523_ peak, but well before the peak for discoidal HDL. This elution pattern suggests that smaller (i.e., <14.1 nm) fmHDL (apo A-I) are more efficient recipients of cellular phospholipid by the ABCA1 pathway than the larger (i.e., >14.1 nm) species.

ABCA1-mediated cholesterol efflux to native spherical HDL is a result of de novo discoidal HDL formation by lipid-poor apo A-I that dissociated from the lipoprotein (43). fmHDL prepared with [^3^H]apo A-I was used to determine whether ABCA1-mediated efflux to fmHDL (apo A-I) was due to apo A-I dissociation from fmHDL, followed by ABCA1-dependent lipidation of the lipid-poor apolipoprotein. BHK-ABCA1 cells were labeled with [^14^C]cholesterol, treated with vehicle or mifepristone, and exposed to fmHDL ([^3^H]apo A-I) or medium without a cholesterol acceptor (Fig. 6B). In parallel, fmHDL ([^3^H]apo A-I) was incubated in tissue culture flasks without cells to produce a no-cells control. BHK-ABCA1 cells concurrently form two sizes of discoidal HDL: 10–12 nm and 7–8 nm (Fig. 6A) (40). [^3^H]apo A-I dissociation from fmHDL with subsequent formation of discoidal HDL would increase the amount of [^3^H] in the 10–12 nm and 7–8 nm elution fractions and correspondingly reduce its content in the fractions overlapping with the fmHDL A_523_ peak. The 7–8 nm fractions of fmHDL exposed to vehicle-treated or ABCA1-expressing cells or cell-free flasks did not differ in the amount of [^3^H]apo A-I (Fig. 6B). The 10–12 nm fractions of fmHDL exposed to ABCA1-expressing cells reproducibly contained slightly more [^3^H]apo A-I (∼6% of the whole profile [^3^H]apo A-I) than the corresponding fractions of the vehicle-treated and no-cells controls. The source of this additional [^3^H]apo A-I in the 10–12 nm region was not apparent. The [^3^H]apo A-I traces for fmHDL (apo A-I) exposed to vehicle-treated cells and fmHDL (apo A-I) exposed ABCA-expressing cells tracked each other closely in the 17.7–12.5 nm size region that includes the fmHDL (apo A-I) A_523_ and cholesterol peaks (Fig. 6B). This implies that fmHDL particles in the 17.7–12.5 nm region did not lose any apo A-I. Thus, the majority of ABCA1-mediated cell cholesterol and phospholipid efflux occurs to the fmHDL holoparticle.

### Cholesterol efflux from primary macrophages to fmHDL (apo A-I) proceeds mainly by the ABCA1-mediated pathway

**Fig. 7.**
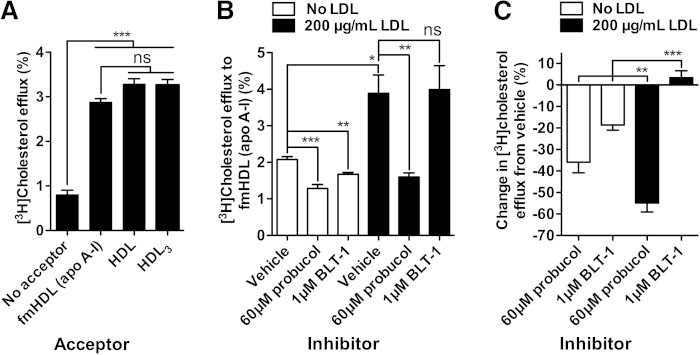
Cholesterol efflux to fmHDL (apo A-I) and native HDL from mouse resident peritoneal macrophages. A: Cholesterol efflux from cholesterol-normal mouse resident peritoneal macrophages to fmHDL (apo A-I), HDL, and HDL_3_. All three acceptors were added to efflux media at 22 μg/ml total protein. B: Cholesterol efflux to fmHDL (apo A-I) by all cholesterol efflux pathways or after inhibition of the ABCA1 (probucol) and SR-BI (BLT-1) pathways from cholesterol-normal and LDL-C-loaded mouse resident peritoneal macrophages. Protein concentrations are the same as in (A). C: Contribution of the ABCA1 and SR-BI pathways to total cholesterol efflux to fmHDL (apo A-I) from macrophages that were or were not loaded with LDL-C. The percent efflux with vehicle was subtracted from the percent efflux with probucol or BLT-1. Then the difference was divided by the percent efflux from vehicle and multiplied by 100. Values are mean ± SEM; n = 6. ***P < 0.001; **P < 0.01; *P < 0.1; ns, not significant.

Murine macrophages simultaneously express and use all three pathways of cholesterol efflux in addition to releasing cholesterol by unmediated diffusion (6, 16). Radiolabeled murine resident peritoneal macrophages were exposed to fmHDL (apo A-I), HDL or HDL_3_ (all at 22 μg/ml total protein). The magnitudes of cholesterol efflux to all three acceptors did not differ significantly, indicating that fmHDL (apo A-I) functioned just as well as native HDL (**Fig. 7A**).

Probucol, an inhibitor of ABCA1 (44), and BLT-1 (42) were used to ascertain the extent to which the ABCA1 and SR-BI pathways, respectively, contributed to macrophage cholesterol efflux to fmHDL (apo A-I). Probucol did not inhibit SR-BI- or ABCG1-mediated cholesterol efflux in BHK-SR-BI and BHK-ABCG1 cells, respectively, validating its specificity for ABCA1 (Fig. 4C). Radiolabeled murine resident peritoneal macrophages were incubated with LDL-free or LDL-containing media, treated with probucol, BLT-1, or vehicle, and exposed to fmHDL (apo A-I) (0.236 μM) in the presence of the inhibitors/vehicles. In macrophages incubated with LDL-free medium, the ABCA1-mediated pathway (i.e., probucol sensitive efflux) accounted for 36 ± 12% of the total cholesterol efflux, whereas the SR-BI-mediated pathway (i.e., BLT-1 sensitive efflux) accounted for 19 ± 6% of the total efflux (Fig. 7B, C). The remainder of cellular cholesterol release was likely due to a combination of simple and ABCG1-facilitated diffusion.

Loading macrophages with cholesterol by incubation with LDL caused cholesterol efflux to fmHDL (apo A-I) to double (Fig. 7B). There was also a significant change in the relative contributions of the ABCA1 and SR-BI pathways (Fig. 7B, C). The contribution of the ABCA1 pathway increased to 54 ± 10% of the total cholesterol efflux, whereas the SR-BI pathway no longer contributed to cholesterol release. Together, these findings show that fmHDL (apo AI) can support the ABCA1 cholesterol efflux pathway in primary macrophages.

## DISCUSSION

Macrophage HDL-C flux, the net transport of macrophage cholesterol in HDL to the liver for secretion, is a critical atheroprotective factor and a promising target for therapeutic intervention (5). One approach that has been intensely explored for enhancing the rate of this process is infusion of various formulations of rdHDL, a synthetically prepared precursor of mature HDL (13, 14). Recently, a rdHDL formulation, CER-001, did not meet prespecified endpoints for improvement in the atheroma volume in a prospective double-blinded randomized trial (45). However, another formulation, CSL112, dramatically stimulated the ABCA1 pathway in an animal model and in human subjects (46, 47). A drawback of rdHDL is that it accepts cholesterol from many cell types nonspecifically (13, 14). In this way, rdHDL is similar to native HDL, which also does not distinguish between cholesterol sources (16). Given that macrophages contribute a relatively small amount of lipid to HDL (48, 49), the bulk of cholesterol that rdHDL receives likely comes from nonmacrophage donors. Furthermore, like native HDL, rdHDL can release cholesterol to proatherogenic lipoproteins by either diffusion or via cholesteryl ester transfer protein (17). Several investigations reported increases in the levels of proatherogenic LDL-C and VLDL-C following rdHDL administration (19–21). Thus, further research is required to design a discriminating therapeutic that avoids the promiscuity of rdHDL and native HDL and targets macrophage cholesterol specifically.

Gold NPs are a flexible platform on which to assemble requisite components. fmHDL contains phospholipid and apo A-I, but other elements can also be readily added (15). In terms of size and surface charge, fmHDL and native HDL are very similar [Table 1 (50, 51)]. However, the choice of an Au NP template renders fmHDL structurally and compositionally different from its native counterpart [Table 1 (51)]. The key goal of this work was to demonstrate that these differences were not detrimental to fmHDL’s ability to participate in cellular cholesterol efflux.

Cells release cholesterol by four well-documented processes: unmediated diffusion and ABCG1-, SR-BI-, and ABCA1-mediated pathways (6, 16). Unmediated (also called aqueous) diffusion involves cholesterol desorption from the donor, diffusion through the water milieu, and collision with and incorporation into the acceptor (16). ABCG1 and SR-BI raise cell cholesterol diffusion by increasing its desorption rate (16). The ABCA1 pathway is mechanistically different from the diffusion-based pathways. ABCA1 mediates the assembly of discoidal HDL from cellular lipids and lipid-poor apo A-I. Cholesterol released by this pathway resides in newly-formed HDL (16). Cholesterol diffusion, whether mediated or unmediated, proceeds down a concentration gradient to any suitable acceptor. fmHDL (apo A-I), fmHDL (apo A-I-SH) and fmHDL (no apo A-I) all supported unmediated diffusion and the ABCG1 and SR-BI pathways (Figs. 3; 4A, D; 7B).

fmHDL (apo A-I) also supported the ABCA1 pathway (Figs. 5–7). ABCA1-mediated cholesterol efflux requires lipid-poor apo A-I (16), which could dissociate from the fmHDL holoparticle and undergo ABCA1-dependent lipidation. This occurs when ABCA1-expressing cells are exposed to spherical HDL (34, 43). Or, partly-lipidated apo A-I could obtain lipid via ABCA1 while remaining bound to fmHDL. This is the mechanism of cholesterol acquisition by certain artificially produced small discoidal HDL (52). Gel filtration analysis detected minimal dissociation of lipid-poor apo A-I from the fmHDL holoparticle (Fig. 6B). In addition, it revealed preferential incorporation of choline-phospholipid into smaller-sized fmHDL (apo A-I) during ABCA1-mediated lipid efflux (Fig. 6A). These observations suggest that apo A-I on fmHDL assumes a partly-lipidated conformation capable of interacting with ABCA1 and accepting cellular lipids (52). Further investigation is necessary to characterize apo A-I structure in fmHDL (apo A-I) and to delineate the interaction between fmHDL (apo A-I) and ABCA1.

Previous ex vivo and in vivo investigations have shown that cholesterol-normal and cholesterol-loaded macrophages employ the cellular cholesterol efflux pathways differently (42, 53). An ideal cholesterol acceptor for improving macrophage HDL-C flux must support the pathways specifically expressed by cholesterol-loaded macrophages. Cholesterol loading increases macrophage efflux by as much as 2-fold and reduces the importance of the SR-BI pathway, while raising the contribution of ABCA1 (42, 53). In line with the previous findings, LDL-C loading of mouse resident peritoneal macrophages approximately doubled macrophage cholesterol efflux to fmHDL (apo A-I) (Fig. 7). The SR-BI pathway did not contribute to efflux in the LDL-C loaded cells, whereas the contribution of the ABCA1 route to fmHDL (apo A-I)-driven efflux significantly increased with cholesterol enrichment (Fig. 7). This demonstrates that fmHDL (apo A-I) is an excellent acceptor of macrophage cholesterol and accommodates well the efflux pathway preferences of cholesterol-loaded macrophages. The impact of fmHDL (apo A-I) infusion on plasma lipids in vivo is under investigation.

A potential advantage of fmHDL is that it is suitable for further functionalization. One possibility is the inclusion of macrophage-specific markers, which would impart fmHDL with enhanced targeting properties (54). In addition to its role in macrophage HDL-C flux, HDL performs many other functions and serves as a carrier of bioactive compounds such as bioactive lipids and short interfering RNAs (35, 55–57). Thus, another possibility is to modify fmHDL particles to carry health-promoting factors (58). In summary, fmHDL (apo A-I) is a fmHDL that supports cholesterol efflux by all documented cell cholesterol efflux pathways and closely accommodates the cholesterol efflux pathway preferences of cholesterol-loaded macrophages. It is a promising therapeutic for increasing HDL-CEC from macrophages and promoting macrophage HDL-C flux with potential advantages over infusable rdHDL.
